# PReS-FINAL-2078: Single nucleotide polymorphisms of toll like receptors 2 and 4 in enthesitis related arthritis and oligo and polyarticular juvenile idiopathic arthritis

**DOI:** 10.1186/1546-0096-11-S2-P90

**Published:** 2013-12-05

**Authors:** M Perica, M Perić, M Vidović, L Tambić Bukovac, L Lamot, J Barbić, M Harjaček

**Affiliations:** 1 Department of Rheumatology, Children's Hospital Srebrnjak, Zagreb, Croatia; 2 Institute of Public Health, Osijek, Croatia; 3 University of Osijek, School of Medicine, Osijek, Croatia

## Introduction

Single nucleotide polymorphisms of Toll like receptors modify cellular immune response and induce pro-inflammatory cytokine production and therefore could be associated with enthesitis related arthritis (ERA) and/or oligoarticular and polyarticular juvenile idiopathic arthritis (JIA).

## Objectives

To determine whether polymorphisms of TLR2 and TLR4 influence susceptibility to ERA or JIA.

## Methods

DNA was extracted from blood samples of 19 ERA patients, 10 patients with oligoarticular or polyarticular JIA and 40 healthy controls, all diagnosed according to ILAR criteria. Polymorphisms of the TLR2 (Arg753Gln) and TLR4 (Asp299Gly, Thr399Ile) were determined using real time and multiplex PCR.

## Results

All JIA patients were carriers of wild type allele for all three polymorphisms. Regarding Arg753Gln polymorphism of TLR2, only one patient with ERA (5.56%) and 2 healthy controls (5%) were carriers of heterozygous allele. There were no homozygous mutants. All ERA patients had wild type allele for Asp299Gly polymorphism of TLR4. For Thr399Ile polymorphism of TLR4, 21.05% ERA patients were heterozygous (CT variant), and none of the ERA patients was homozygous (TT variant), (CC vs CT variant in ERA OR 3.7500, 95% CI 1.0498-13.3952, p 0.0419, CC vs TT variant OR 31.0000, 95% CI 1.7309-555.1867, p 0.0197). In group of healthy controls, TLR4 polymorphisms Asp299Gly and Thr399Ile were in linkage disequilibrium; 2 controls were hetrozygous and 6 homozygous variant carriers for both polymorphisms, whereas linkage disequilibrium was not found among patient groups (CC vs CT in controls OR 16.0000, 95% CI 3.5905-71.2994, p 0.0003, CC vs TT OR 5.3333, 95% CI 2.0099-14.1524, p 0.1944).

## Conclusion

Polymorphisms of TLR2 and TLR4 are not associated with oligoarticular/polyarticular JIA. There was also no evidence that variants of TLR are major risk factors for ERA, however, lack of susceptibility should be confirmed on larger group of patients, since four out of 19 patients (21,05%) were heterozygous. A study on larger cohort is currently ongoing.

## Disclosure of interest

None declared.

